# Chiral isothiourea-catalyzed kinetic resolution of 4-hydroxy[2.2]paracyclophane

**DOI:** 10.3762/bjoc.17.68

**Published:** 2021-04-08

**Authors:** David Weinzierl, Mario Waser

**Affiliations:** 1 Institute of Organic Chemistry, Johannes Kepler University Linz, Altenbergerstrasse 69, 4040 Linz, Austria

**Keywords:** acylation, kinetic resolution, nucleophilic catalysis, paracyclophanes, planar chirality

## Abstract

We herein report a method for the kinetic resolution of racemic 4-hydroxy[2.2]paracyclophane by means of a chiral isothiourea-catalyzed acylation with isobutyric anhydride. This protocol allows for a reasonable synthetically useful *s*-factor of 20 and provides a novel entry to obtain this interesting planar chiral motive in an enantioenriched manner.

## Introduction

Substituted [2.2]paracyclophanes are fascinating planar chiral molecules [1–12] which have been systematically investigated since Brown and Farthing discovered the formation of the unsubstituted and achiral parent [2.2]paracyclophane (**1**) via gas phase pyrolysis of *para*-xylene in 1949 [5]. Over the years, these compounds established themselves as a unique class of “bent and battered” [6] strained molecules with remarkable chemical and physical properties [1–4,7–9]. Besides their potential applications in material and polymer chemistry [1–2,7–9], these planar chiral molecules have been very successfully used in asymmetric catalysis [3–4,10–12]. Accordingly, the development of methods for the asymmetric synthesis of enantiomerically pure, or at least enantiomerically enriched, derivatives that can be utilized as building blocks for more demanding ligands and catalysts became a task of high importance. Thus, several strategies to access enantioenriched [2.2]paracyclophanes have been reported, either relying on classical resolution approaches or, more recently, making use of asymmetric catalysis to carry out kinetic resolutions of easily accessed racemic precursors [3–4,13–15]. 4-Hydroxy[2.2]paracyclophane (**2**) is one of the commonly used building blocks, which is easily accessible in a racemic manner starting from **1** according to nowadays well-established procedures [16–18]. Over the last decades, it was shown that enantioenriched **2** may serve as a valuable building block to access more advanced chiral cyclophane ligands and catalysts [3–4,19–22] and therefore its asymmetric synthesis became an important task [3–4,18–27]. Several strategies to access **2** in an enantioenriched fashion have been developed. One commonly used method relies on the resolution of 4-formyl[2.2]paracyclophane via formation of a chiral Schiff base first, followed by a subsequent Dakin-type oxidation to alcohol **2** [18]. Alternatively, the direct resolution of *rac*-**2** via transformation into diastereomers by esterification with chiral acid chlorides [19–20] as well as the kinetic resolution (KR) of racemic esters of **2** via an enzymatic hydrolysis [25–27] were very successfully used to access enantioenriched **2**. Recently, Akiyama and co-workers reported the kinetic resolution of *rac*-PHANOL (4,12-dihydroxy[2.2]paracyclophane) by means of a chiral phosphoric acid-catalyzed esterification with achiral anhydrides [28]. This method allowed for high *s*-factors but was unfortunately not satisfyingly applicable to *rac*-4-hydroxy[2.2]paracyclophane (*rac*-**2**) [28].

**Scheme 1:**
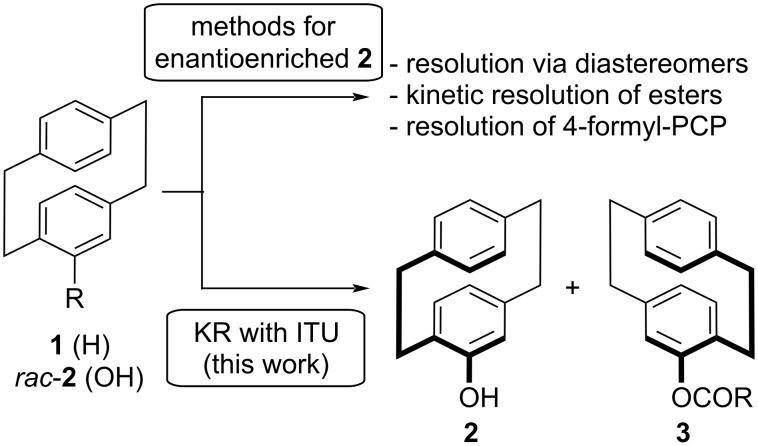
Overview about established methods to access enantioenriched **2** and the herein investigated kinetic resolution (KR) with chiral isothiourea (ITU) catalysts.

Considering the interest in compound **2**, we thus thought about developing an alternative organocatalytic kinetic resolution protocol to control the esterification of *rac*-**2**. Chiral isothioureas (ITUs) emerged as easily available and powerful catalysts for numerous applications [29–32] and have been very successfully used for the kinetic resolution of different racemic alcohols [33–37]. Inspired by this unique catalysis potential, we therefore became interested in testing those chiral catalysts for the, to the best of our knowledge, so far not investigated acylative kinetic resolution of 4-hydroxy[2.2]paracyclophane (**2**, Scheme 1).

## Results and Discussion

**Scheme 2:**
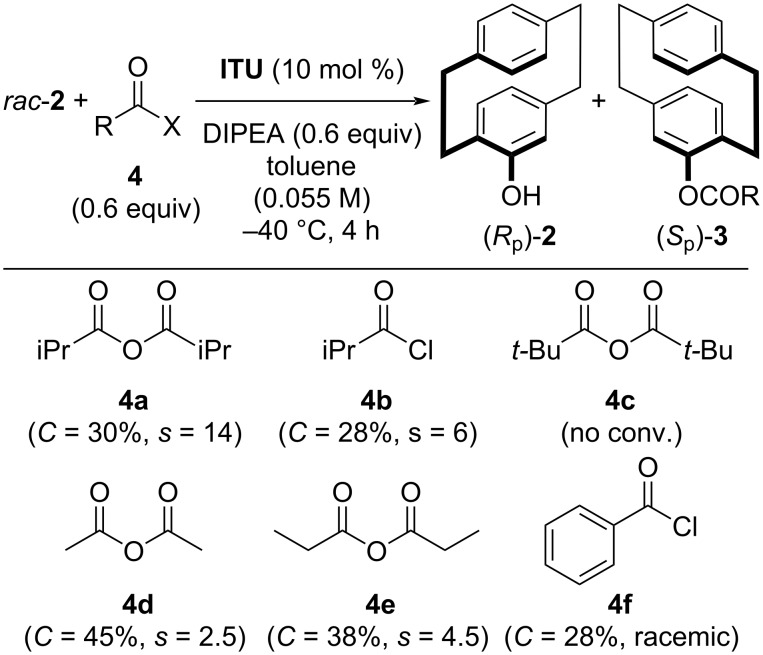
Use of alternative acylating agents **4** for the kinetic resolution of *rac*-**2**.

BTM (**ITU 1** [33]) and HyperBTM (**ITU 2** [38]) are amongst the most commonly used chiral ITUs and these nowadays commercially available catalysts were used to optimize the resolution of *rac*-**2** with isobutyric anhydride (**4a**) (Table 1 gives an overview of the most significant results obtained in this screening). Anhydride **4a** was chosen in a first instance as it proved successful in previous acylative resolutions reported by others [28,33–34,36–37] but we later on also tested other anhydrides and acid chlorides (vide infra, Scheme 2). First experiments with 10 mol % BTM (**ITU 1**) carried out in CHCl_3_ or toluene at room temperature (Table 1, entries 1 and 2) proved the general feasibility of this concept, resulting in *s*-factors around 6. When lowering the temperature, a slight improvement could be achieved at −15 °C (Table 1, entry 3) but unfortunately **ITU 1** performed less selective at −78 °C (Table 1, entry 4). Instead, (2*S*,3*R*)-HyperBTM (**ITU 2**) resulted in an enhanced selectivity with *s* = 14.5 at −78 °C but conversion was relatively slow (Table 1, entry 5). Gratefully however, the obtained *s*-factor was almost the same at −40 °C and a reasonable conversion of around 30% could be observed after 4 h reaction time (Table 1, entry 6). Varying solvent and concentration at −40 °C next showed that toluene allows for higher selectivities than CHCl_3_ (compare Table 1, entries 6 and 7), while the use of other solvents like CH_2_Cl_2_ and THF resulted in almost no product formation and no reasonable selectivities (not mentioned in Table 1). In addition, higher concentrations lead to notably lower selectivities (Table 1, entry 9), while more diluted conditions did not allow for a significant improvement of the *s*-factor anymore (Table 1, entry 8). Lowering the catalyst loading from 10 to 5 mol % allowed for a similar conversion, but resulted in a slightly reduced selectivity (Table 1, entry 10).

**Table 1:** Identification of the optimum catalyst and best conditions for the resolution of *rac*-**2** with anhydride **4a**^a^.

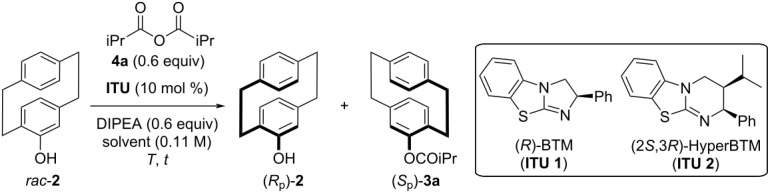								

Entry	ITU	Solvent	*T* [°C]	*t* [h]	Conv. (*C*) [%]^b^	ee (**2**) [%]^c,d^	ee (**3a**) [%]^c^	*s*^e^

1	**ITU 1**	CHCl_3_	25	1	41	42	60	6
2	**ITU 1**	toluene	25	1	38	39	64	6.5
3	**ITU 1**	toluene	−15	1	34	38	74	10
4	**ITU 1**	toluene	−78	1	15	13	74	7.5
5	**ITU 2**	toluene	−78	1	16	16	85	14.5
6	**ITU 2**	toluene	−40	4	33	40	81	14
7	**ITU 2**	CHCl_3_	−40	4	45	55	67	9
8	**ITU 2**	toluene (0.055 M)	−40	4	30	35	82	14.5
9	**ITU 2**	toluene (0.22 M)	−40	4	36	32	75	9.5
10^f^	**ITU 2**	toluene	−40	4	30	34	79	12
11^g^	**ITU 2**	toluene	−40	22	57	94 (39%)^h^	71 (53%)^h^	20

^a^All reactions were carried out using 0.1 mmol *rac*-**2** and 0.06 mmol **4a** in the presence of 0.06 mmol Hünig’s base (diisopropylethylamine, DIPEA) and 10 mol % ITU in the indicated solvent (0.11 M with respect to **2**) unless otherwise stated; ^b^determined by ^1^H NMR of the crude product; isolated yields of **2** and **3** were almost quantitative in all cases; ^c^determined by HPLC using a chiral stationary phase; ^d^absolute configuration of recovered **2** was assigned to be (*R*_p_) by comparison of its (+)-optical rotation with previous reports [20,26,39]; ^e^the *s*-factor was calculated from the ee of recovered **2** and/or the ee of ester **3** [40–43]; ^f^using 5 mol % **ITU 2**; ^g^using 1.1 equiv of **4a**; ^h^isolated yield when carried out on 1 mmol *rac*-**2** scale.

At this point, we decided to screen other anhydrides and acid chlorides **4**, but, as outlined in Scheme 2, the initially used isobutyric anhydride **4a** clearly outperformed its analogous acid chloride **4b**, as well as the other derivatives **4c**–**f**.

Finally, the resolution of *rac*-**2** was run for 22 h in the presence of 10 mol % HyperBTM (**ITU 2**) with 1.1 equivalents of anhydride **4a** (instead of the previously used 0.6 equiv; Table 1, entry 11). Under these conditions it was possible to achieve a conversion of slightly above 50% combined with good enantioselectivities for both, the recovered alcohol **2** and the ester **3a** (*s* = 20). With these optimum conditions the resolution was also successfully carried out on 1 mmol scale, resulting in an identical conversion and *s*-factor (*s* = 20; *C* = 57%) and allowing for the isolation of (*R*_p_)-**2** in 39% yield (94% ee) and (*S*_p_)-**3a** in 53% yield (71% ee) (Table 1, entry 11). Mechanistically, this resolution process should proceed via the well-understood formation of a chiral acyl-transfer species between the isothiourea catalyst **ITU 2** and the anhydride **4a** [33–37], which then allows for the resolution of the enantiomers of alcohol **2**. Unfortunately, however, the true nature of this enantiodiscriminating step has not yet been elucidated and will require detailed computational studies.

## Conclusion

In conclusion, we identified conditions that allow for the kinetic resolution of racemic 4-hydroxy[2.2]paracyclophane (**2**) by means of an acylation with isobutyric anhydride (**4a**) in the presence of the chiral isothiourea catalyst HyperBTM (**ITU 2**). The reaction can be carried out with an *s*-factor around 20 and allows for the isolation of recovered (*R*_p_)-**2** and ester (*S*_p_)-**3a** with reasonable enantiomeric excesses around 90%, depending on the conversion. These two compounds can easily be separated by silica gel column chromatography in almost quantitative yields, thus providing a novel entry to obtain these interesting planar chiral motives in an enantioenriched manner.

## Experimental

### General details

^1^H- and ^13^C NMR spectra were recorded on a Bruker Avance III 300 MHz spectrometer with a broad band observe probe and a sample changer for 16 samples. NMR spectra were referenced on the solvent peak and chemical shifts are given in ppm.

High-resolution mass spectra were obtained using a Thermo Fisher Scientific LTQ Orbitrap XL with an Ion Max API Source. Analyses were made in the positive ionization mode if not otherwise stated. HPLC was performed using a Thermo Scientific Dionex Ultimate 3000 system with diode array detector with a CHIRAL ART Cellulose-SB stationary phase. Optical rotations were recorded on a Schmidt + Haensch Polarimeter Model UniPol L1000 at 589 nm.

All chemicals were purchased from commercial suppliers and used without further purification unless otherwise stated. *rac*-**2** was prepared from **1** according to a previously published procedure [16].

### Optimized procedure for the KR of *rac*-**2**

Racemic 4-hydroxy[2.2]paracyclophane (*rac*-**2**; 250 mg; 1.115 mmol) and HyperBTM (**ITU 2;** 35 mg; 10 mol %) were dissolved in dry toluene (10 mL) in a Schlenk flask (Ar atmosphere), followed by the addition of Hünig’s base (DIPEA; 118 µL; 0.67 mmol; 0.6 equiv). The solution was then cooled to −40 °C and isobutyric anhydride (**4a**; 208 µL; 1.226 mmol; 1.1 equiv) was added and the mixture was stirred at −40 °C for 22 h. The reaction was quenched by addition of MeOH. The crude product was filtered over Na_2_SO_4_ and the solvent removed in vacuum. Recovered alcohol **2** and ester **3a** were separated by silica gel column chromatography (heptanes/ethyl acetate 10:1), yielding (*S*_p_)-**3a** in 53% (175 mg) and (*R*_p_)-**2** in 43% (98 mg) (39%).

(*R*_p_)-**2a**: Analytical data match those reported in literature [18–20,26,28,39]. TLC (heptanes/ethyl acetate 10:1; *R*_f_ = 0.11). [α]_D_^24^ 14.1 (*c* 1, CH_2_Cl_2_, 92% ee) and 12.1 (*c* 1, CHCl_3_, 92% ee); ^1^H NMR (300 MHz, CDCl_3_, 298.0 K) δ/ppm 7.00 (dd, *J* = 8, 1.8 Hz, 1H), 6.55 (dd, *J* = 8, 1.8 Hz, 1H), 6.45 (dd, *J* = 8, 1.8 Hz, 1H), 6.41–6.37 (m, 2H), 6.26 (dd, *J* = 8, 1.8 Hz, 1H), 5.54 (d, *J* = 1.6 Hz, 1H), 4.42 (s, 1H), 3.37–3.29 (m, 1H), 3.14–3.02 (m, 4H), 2.98–2.85 (m, 2H), 2.71–2.60 (m, 1H); ^13^C NMR (75 MHz, CDCl_3_, 298.0 K) δ/ppm 153.8 (1C, CAr), 142.1 (1C, CAr), 139.8 (1C, CAr), 139.0 (1C, CAr), 135.6 (1C, CAr), 133.8 (1C, CAr), 132.9 (1C, CAr), 132.0 (1C, CAr), 128.1 (1C, CAr), 125.6 (1C, CAr), 125.2 (1C, CAr), 122.7 (1C, CAr), 35.4 (1C, -CH_2_), 34.9 (1C, -CH_2_), 34.0 (1C, -CH_2_), 32.2 (1C, -CH_2_); HRMS (ESI) *m*/*z*: calcd for [C_16_H_16_O + H]^+^, 225.1274; found, 225.1280, HPLC: YMC Chiral ART Cellulose-SB, *n*-hexane/iPrOH 3:1, 1 mL/min, 10 °C; *t*_R_ = 6.4 min [*S*_p_; minor], 7.2 min [*R*_p_; major].

(*S*_p_)-**3a**: Analytical data match those reported in literature [28]. TLC (heptanes/ethyl acetate 10:1; *R*_f_ = 0.33). [α]_D_^24^ 27.5 (*c* 1.0, CHCl_3_, 82% ee); mp 80–82 °C; ^1^H NMR (300 MHz, CDCl_3_, 298.0 K) δ/ppm 6.91 (dd, *J* = 7.8, 1.8 Hz, 1H), 6.56–6.43 (m, 5H), 6.00 (d, *J* = 1.7 Hz, 1H), 3.17–2.94 (m, 7H), 2.93–2.79 (m, 1H), 2.73–2.64 (m, 1H), 1.42 (d, *J* = 7 Hz, 3H), 1.38 (d, *J* = 7 Hz, 3H); ^13^C NMR (75 MHz, CDCl_3_, 298.0 K) δ/ppm 174.8 (1C, C=O), 149.1 (1C, CAr), 141.7 (1C, CAr), 139.6 (1C, CAr), 139.3 (1C, CAr), 135.4 (1C, CAr), 133.5 (1C, CAr), 133.1 (1C, CAr), 132.3 (1C, CAr), 131.1 (1C, CAr), 130.1 (1C, CAr), 129.6 (1C, CAr), 128.2 (1C, CAr), 35.4 (1C, -CH_2_), 35.0 (1C, -CH_2_), 34.4 (2C, -CH, -CH_2_), 31.8 (1C, -CH_2_), 19.4 (1C, -CH_3_), 19.1 (1C, -CH_3_); HRMS (ESI) *m*/*z*: calcd for [C_20_H_22_O_2_ + NH_4_]^+^, 312.1958; found, 312.1958, HPLC: YMC Chiral ART Cellulose-SB, *n*-hexane/iPrOH 3:1, 1 mL/min, 10 °C; *t*_R_ = 7.3 min [*R*_p_; minor], 8.4 min [*S*_p_; major].

## Supporting Information

File 1 Copies of NMR spectra and HPLC chromatograms as well as analytical data of esters **3** obtained with the alternative acyl-transfer reagents **4**.
