# Supplementary material for: Chiral isothiourea-catalyzed kinetic resolution of 4-hydroxy[2.2]paracyclophane
Source: Beilstein J Org Chem. 2021 Apr 8;17:800–4. doi: 10.3762/bjoc.17.68 (PMC8042488; doi:10.3762/bjoc.17.68)
Supplement: File 1 — Copies of NMR spectra and HPLC chromatograms as well as analytical data of esters 3 obtained with the alternative acyl-transfer reagents 4. [file Beilstein_J_Org_Chem-17-800-s001.pdf]

BEILSTEIN JOURNAL OF ORGANIC CHEMISTRY

Supporting Information

for

Chiral isothiourea-catalyzed kinetic resolution of 4-hydroxy[2.2]paracyclophane

David Weinzierl and Mario Waser

Beilstein J. Org. Chem. **2021,** *17,* 800–804. doi:10.3762/bjoc.17.68

Copies of NMR spectra and HPLC chromatograms as well as analytical data of esters 3 obtained with the alternative acyl-transfer reagents 4

License and Terms: This is a supporting information file under the terms of the Creative Commons Attribution License (https://creativecommons.org/licenses/by/4.0). Please note that the reuse, redistribution and reproduction in particular requires that the author(s) and source are credited and that individual graphics may be subject to special legal provisions.
The license is subject to the *Beilstein Journal of Organic Chemistry* terms and conditions: (https://www.beilstein-journals.org/bjoc/terms)

1. Asymmetric *O*-acylation reactions:

General procedure for the screening of different acyl-transfer reagents 4:

Racemic 4-hydroxy[2.2]paracyclophane (**2**, 0.1 mmol) and catalyst **ITU 2** (10 mol %) were dissolved in dry toluene (1.5 mL) in a Schlenk flask under argon, before adding Hünig's base (diisopropylethylamine, DIPEA; 0.06 mmol) as a solution in toluene (0.15 mL). This solution was then cooled to −40 °C and a solution of acylating agent **4** (0.6 mmol) in 0.15 mL toluene was added (resulting in a concentration of 0.055 M with respect to **2**) and the mixture stirred for 4 h. The reaction was quenched with MeOH in the cold and allowed to reach room temperature. The crude product was filtered over Na_2SO_4 and the solvents removed in vacuum, before the desired product was purified by column chromatography.

***Analytical data for other O*-acylated paracyclophanes 3**

3b: Following the general procedure using anhydride **4d**, a conversion of 45% of *rac*-**2** was achieved.

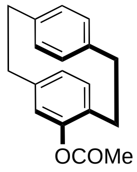

3b

Ester **3b** was obtained as a white solid after silica gel column chromatography using heptanes/ethyl acetate (10:1); ***e.r.*** = 68:32 (s = 2.5); TLC (heptanes/ethyl acetate = 10/1): R_f= 0.21 (UV). Analytical data are in accordance with those reported in literature[1]. $[\alpha]_D^{22}$= 14.1 (c 0.62, CH_2Cl_2, *e.r.* = 68:32); **m.p.** = 125-130°C; **^{1}H-NMR** (300 MHz, $CDCl_3$, 298.0 K): δ / ppm = 6.91 (dd, *J* = 7.8, 1.8 Hz, 1H), 6.56 - 6.43 (m, 5H), 6.01 (d, *J* = 1.7 Hz, 1H), 3.20 - 2.96 (m, 7H), 2.76 - 2.67 (m, 1H), 2.34 (s, 3H); **^{13}C-NMR** (75 MHz, $CDCl_3$, 298.0 K): δ / ppm = 169.0 (1C, C=O), 149.0 (1C, C_{Ar}), 141.7 (1C, C_{Ar}), 139.6 (1C, C_{Ar}), 139.3 (1C, C_{Ar}), 135.4 (1C, C_{Ar}), 133.5 (1C, C_{Ar}) 133.0 (1C, C_{Ar}), 132.3 (1C, C_{Ar}), 131.2 (1C, C_{Ar}), 130.4 (1C, C_{Ar}), 129.6 (1C, C_{Ar}), 128.0 (1C, C_{Ar}), 35.4 (1C, $-CH_2$), 35.0 (1C, $-CH_2$), 34.4 (1C, -CH2), 31.8 (1C, -CH2), 21.3 (1C, $-CH_3$); **HRMS** (ESI) *m/z*: calculated for $[C_{18}H_{18}O_2 + H]^+$: 267.1380; found: 267.1389, **HPLC:** YMC Chiral ART Cellulose-SB, *n*-hexane/*i*-PrOH 3:1, 1 mL/min, 10 °C; t_R = 9.2 min [minor], 11.1 min [major].

3c: Following the general procedure using **4e**, a conversion of 38% of *rac*-**2** was achieved. Ester **3c** was

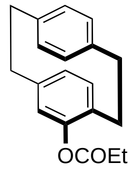

3c

obtained as a white solid after silica gel column chromatography using heptanes/ethyl acetate (10:1); ***e.r.*** = 77:23 (s = 4.5); TLC (heptanes/ethyl acetate = 10/1): R_f = 0.27 (UV). $[\alpha]_D^{22}$= 12.5 (c 0.75, CH_2Cl_2, *e.r.* = 77:23); **m.p.** = 68-70°C; **^{1}H-NMR** (300 MHz, $CDCl_3$, 298.0 K): δ / ppm = 6.91 (dd, *J* = 7.8, 1.8 Hz, 1H), 6.55 - 6.42 (m, 5H), 6.01 (d, *J* = 1.7 Hz, 1H), 3.17 - 2.96 (m, 7H), 2.74 - 2.67 (m, 2H), 2.64 (q, 1H, *J* = 7.5 Hz) 1.35 (t, *J* = 7.5 Hz, 3H); **^{13}C-NMR** (75 MHz, $CDCl_3$, 298.0 K): δ / ppm = 172.3 (1C, C=O), 149.0 (1C, C_{Ar}), 141.7 (1C, C_{Ar}), 139.6 (1C, C_{Ar}), 139.3 (1C, C_{Ar}), 135.4 (1C, C_{Ar}), 133.5 (1C, C_{Ar}), 133.0 (1C, C_{Ar}), 132.4 (1C, C_{Ar}), 131.1 (1C, C_{Ar}), 130.2 (1C, C_{Ar}), 129.6 (1C, C_{Ar}), 128.1 (1C, C_{Ar}), 35.4 (1C, $-CH_2$), 35.0 (1C, $-CH_2$), 34.4 (1C, $-CH_2$), 31.8 (1C, $-CH_2$), 28.1 (1C, $-CH_2$), 9.5 (1C, $-CH_2$); **HRMS** (ESI) *m/z*: calculated for $[C_{19}H_{20}O_2 + H]^+$: 281.1536; found: 281.1541, **HPLC:** YMC Chiral ART Cellulose-SB, *n*-hexane/*i*-PrOH 3:1, 1 mL/min, 10 °C; t_R = 8.7 min [minor], 10.4 min [major].

[1] a) Cipiciani, A.; Fringuelli, F.; Mancini, V.; Piermatti, O.; Pizzo, F. *J. Org. Chem.* **1997**, *62*, 3744-3747; b) Rozenberg, V.; Danilova, T.; Sergeeva, E.; Vorontsov, E.; Starikova, Z.; Lysenko, K.; Belokon, Y. *Eur. J. Org. Chem.* **2000**, 3295–3303; c) Cipiciani, A.; Fringuelli, F.; Mancini, V.; Piermatti, O.; Scappini, A. M.; Ruzziconi, R. *Tetrahedron* **1997**, *53*, 11853–11858.

3d: Following the general procedure, a conversion of 30% of rac-2 was achieved. Ester **3d** was obtained

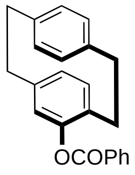

3d

as a white solid after silica gel column chromatography using heptanes/ethyl acetate (10:1). ***rac***; TLC (heptanes/ethyl acetate = 10/1): $\boldsymbol{R_f}$ = 0.30 (UV). **m.p.** = 135-139°C; **^{1}H-NMR** (300 MHz, $CDCl_3$, 298.0 K): δ / ppm = 8.32-8.27 (m, 2H), 7.71-7.66 (m, 1H), 7.61-7.56(m, 2H), 7.04 (dd, *J* = 7.8, 1.8 Hz, 1H), 6.58 - 6.45 (m, 5H), 6.16 (d, *J* = 1.7 Hz, 1H), 3.28 - 3.18 (m, 1H), 3.15 - 2.97 (m, 6H), 2.79 - 2.69 (m, 1H); **^{13}C-NMR** (75 MHz, $CDCl_3$, 298.0 K): δ / ppm = 164.5 (1C, C=O), 149.2 (1C, C_{Ar}), 141.9 (1C, C_{Ar}), ,139.7 (1C, C_{Ar}), 139.4 (1C, C_{Ar}), 135.5 (1C, C_{Ar}), 133.7 (1C, C_{Ar}), 133.6 (1C, C_{Ar}), 133.2 (1C, C_{Ar}), 132.4 (1C, C_{Ar}), 131.3 (1C, C_{Ar}), 130.3 (1C, C_{Ar}), 130.2 (2C, C_{Ar}), 130.0 (1C, C_{Ar}), 129.7 (1C, C_{Ar}), 128.9 (2C, C_{Ar}), 128.3 (1C, C_{Ar}), 35.4 (1C, -CH_2), 35.0 (1C, -CH_2), 34.7 (1C, -CH_2), 32.0 (1C, -CH_2); **MS** (ESI) *m*/*z*: calculated for $[C_{23}H_{20}O_2 + H]^+$: 329.1536; found: 329.1531, **HPLC:** YMC Chiral ART Cellulose-SB, *n*-hexane/*i*-PrOH 3:1, 1 mL/min, 10 °C; t_R = 9.0, 9.7 min;

2. NMR spectra of products 3

NMR-spectra of **3a**

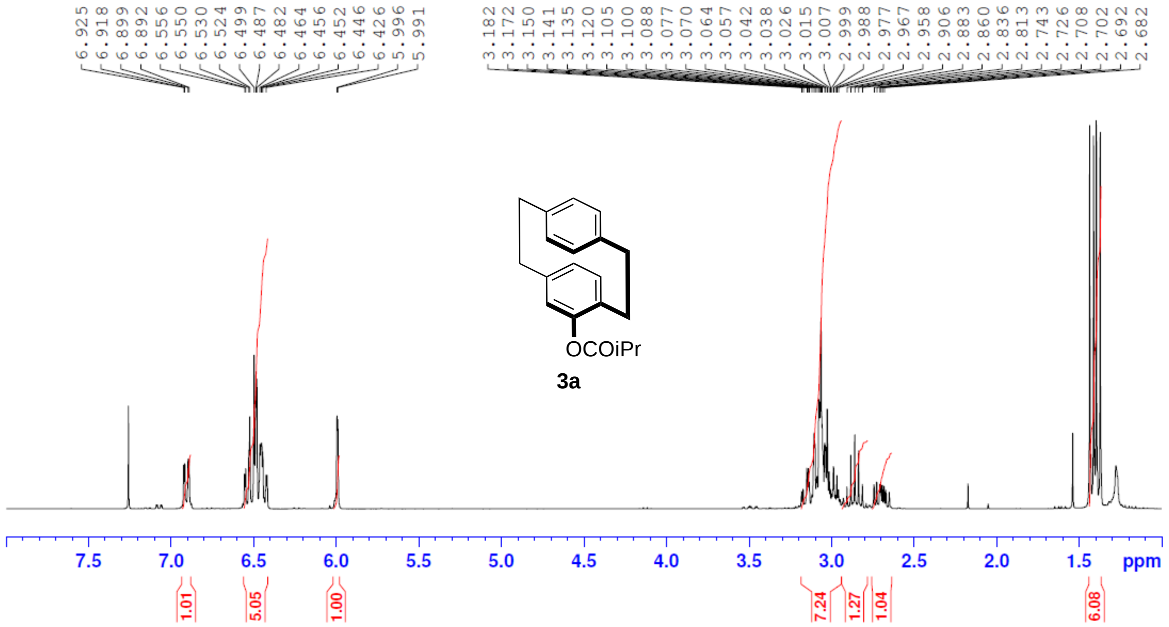

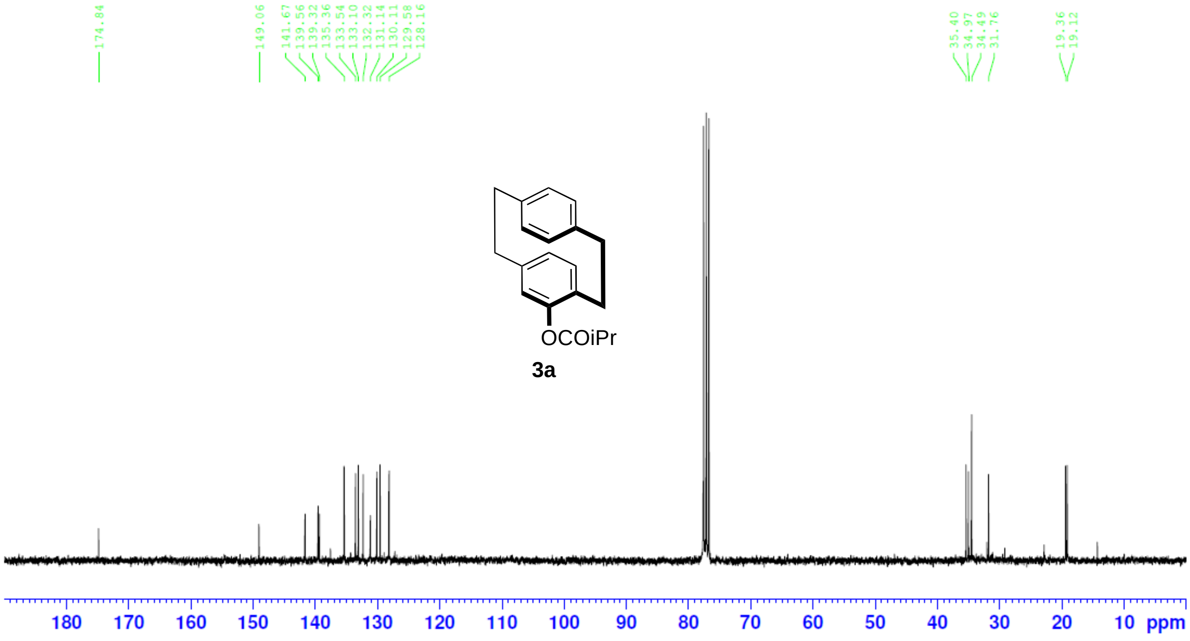

NMR-spectra of **3b**

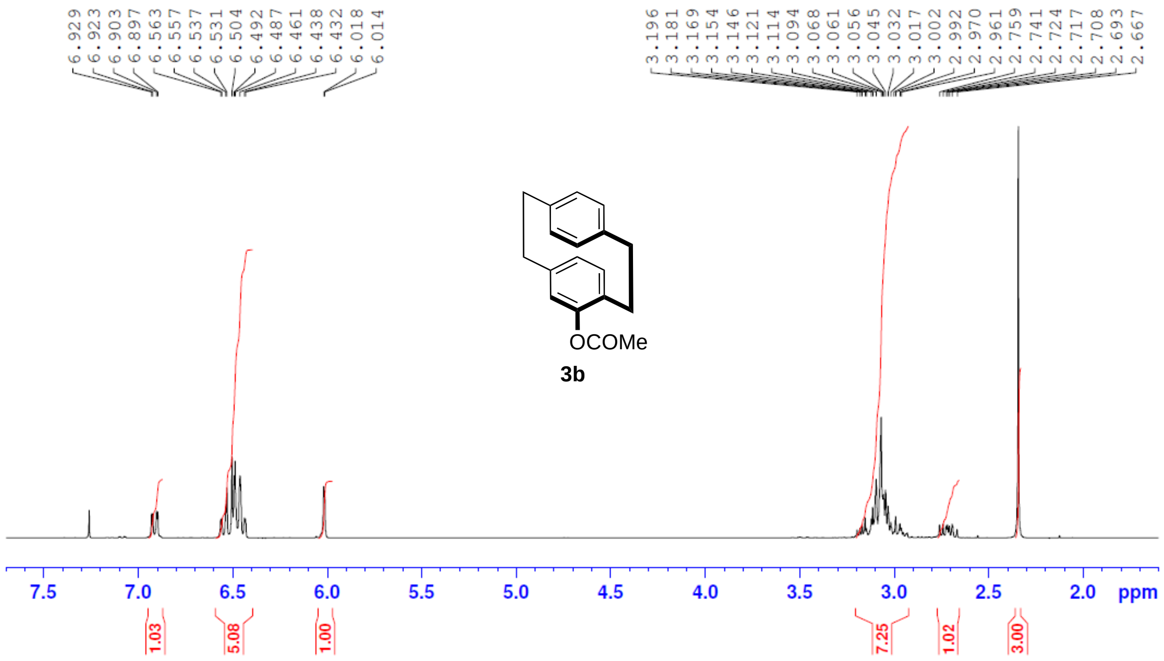

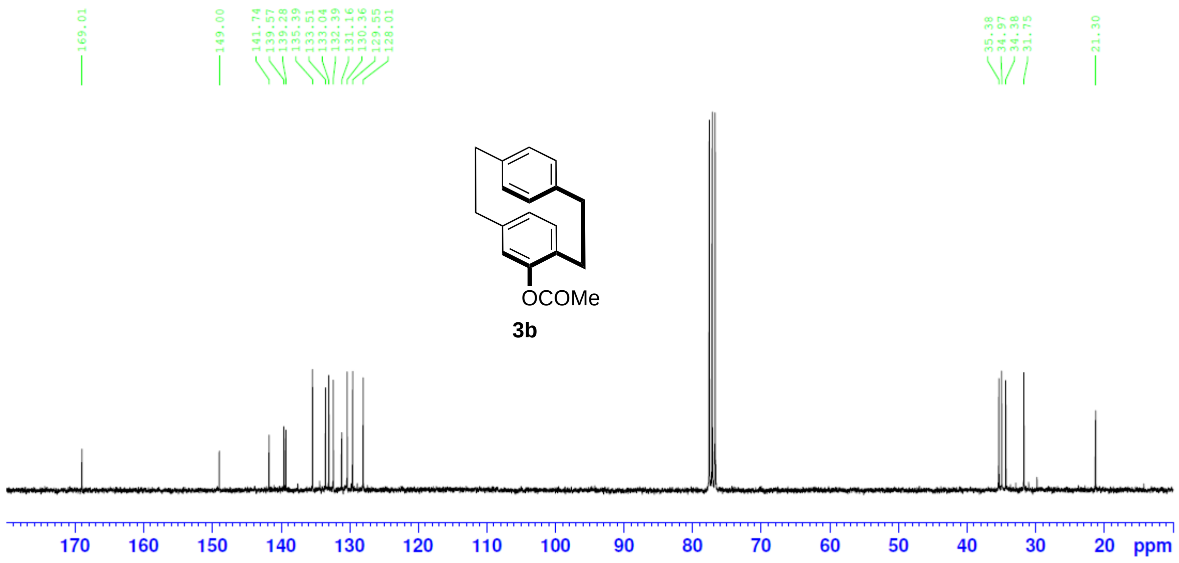

NMR-spectra of **3c**

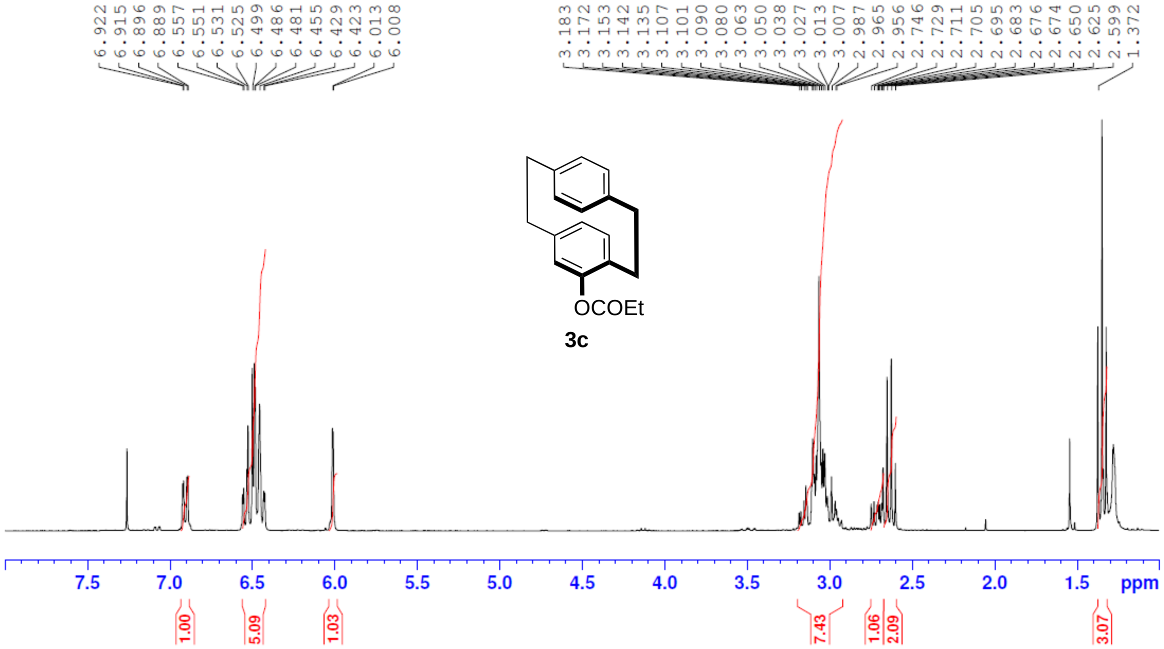

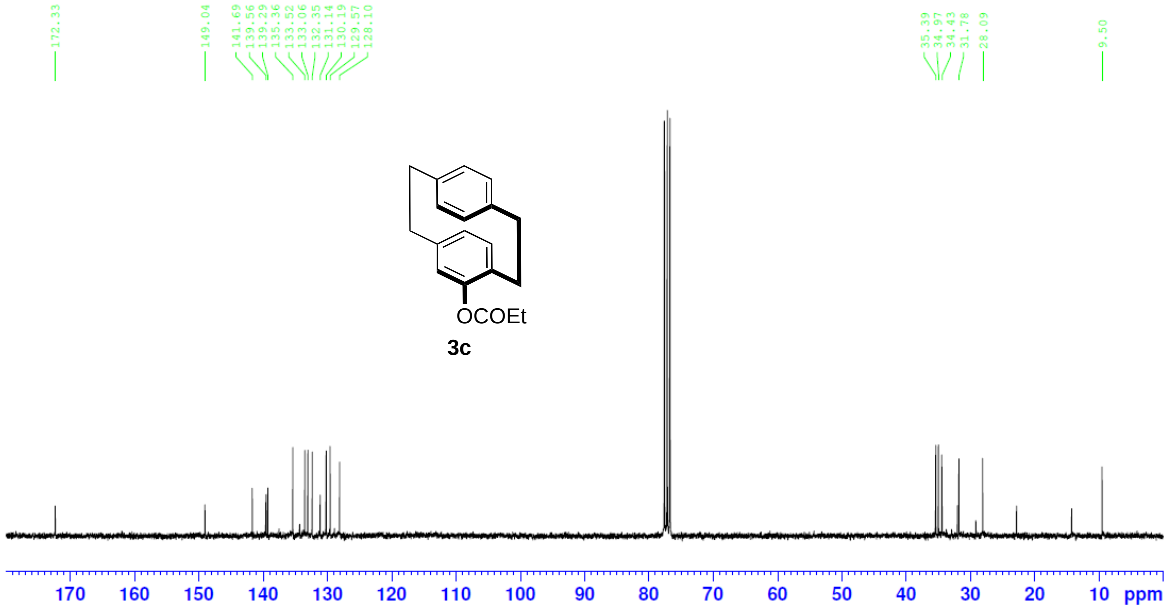

NMR-spectra of **3d**

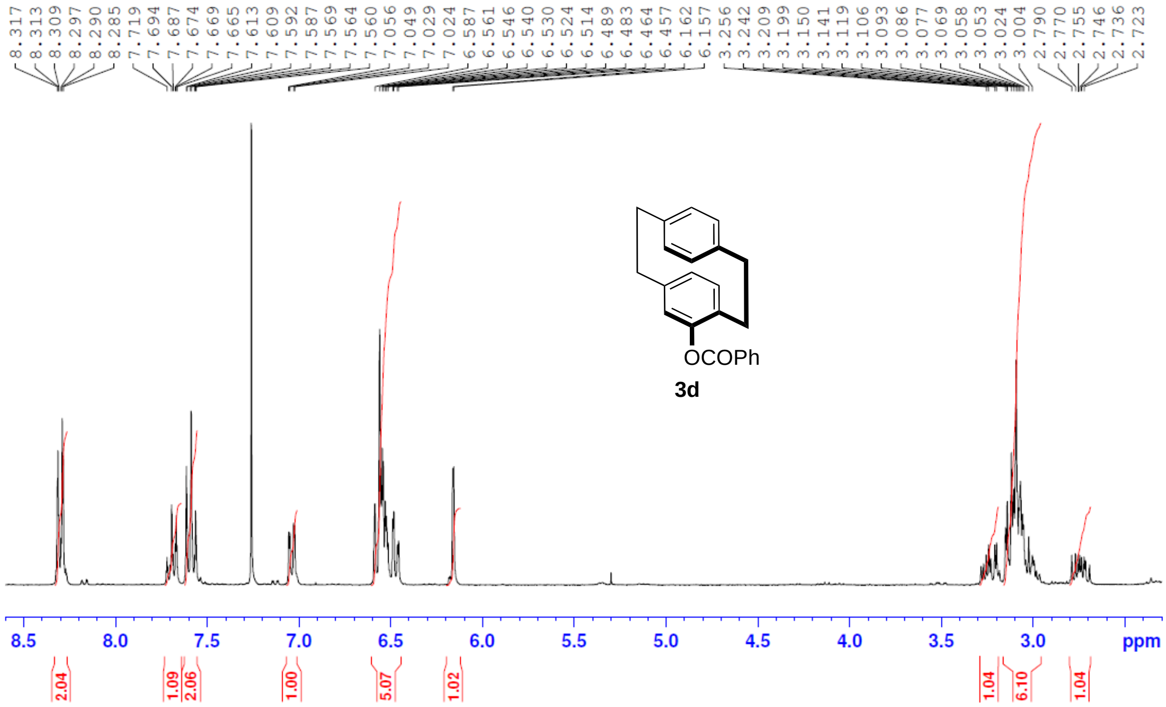

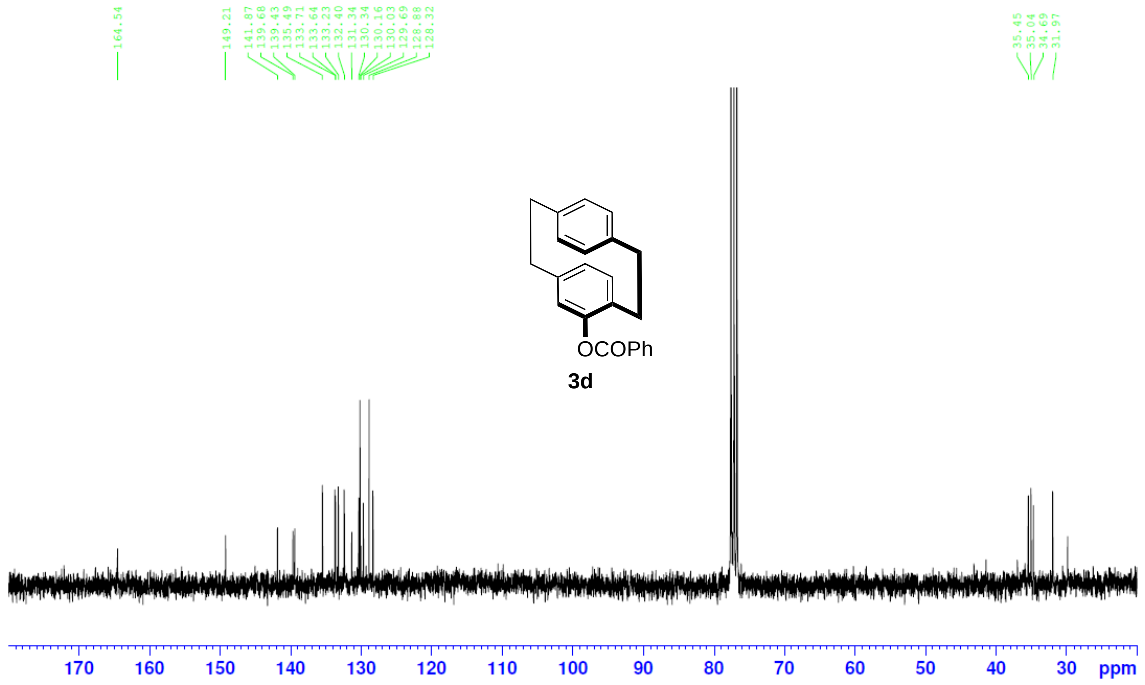

3. HPLC Chromatograms

HPLC chromatogram of (*rac*)-**2**:

Chromatogram

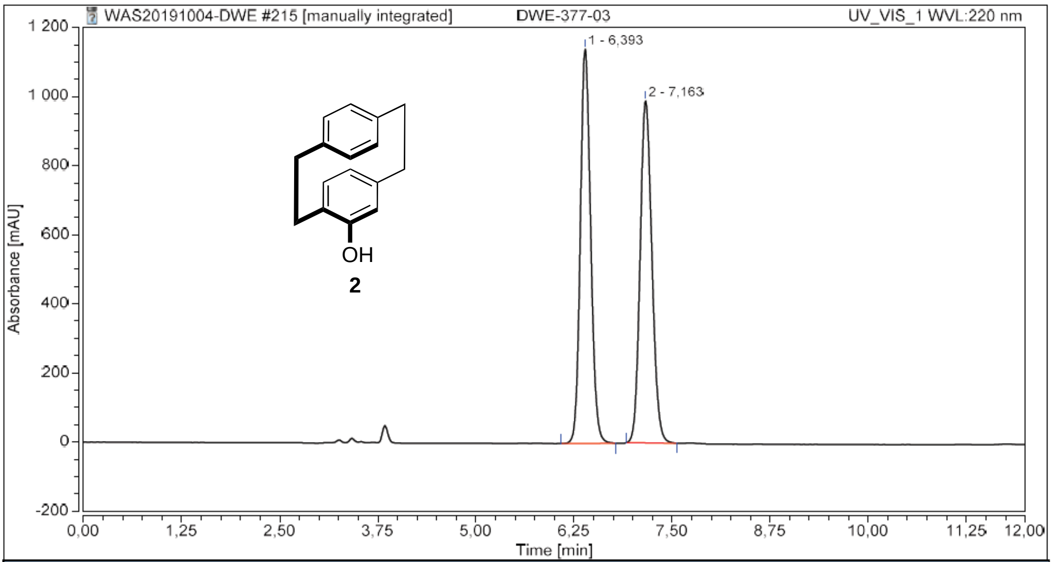

Integration Results

No.	Peak Name	Retention Time min	Area mAU*min	Height mAU	Relative Area %	Relative Height %	Amount n.a.
1		6,393	173,282	1141,130	50,27	53,56	n.a.
2		7,163	171,449	989,582	49,73	46,44	n.a.
**Total:**			**344,731**	**2130,713**	**100,00**	**100,00**	

HPLC chromatogram of enantioenriched **2**:

Chromatogram

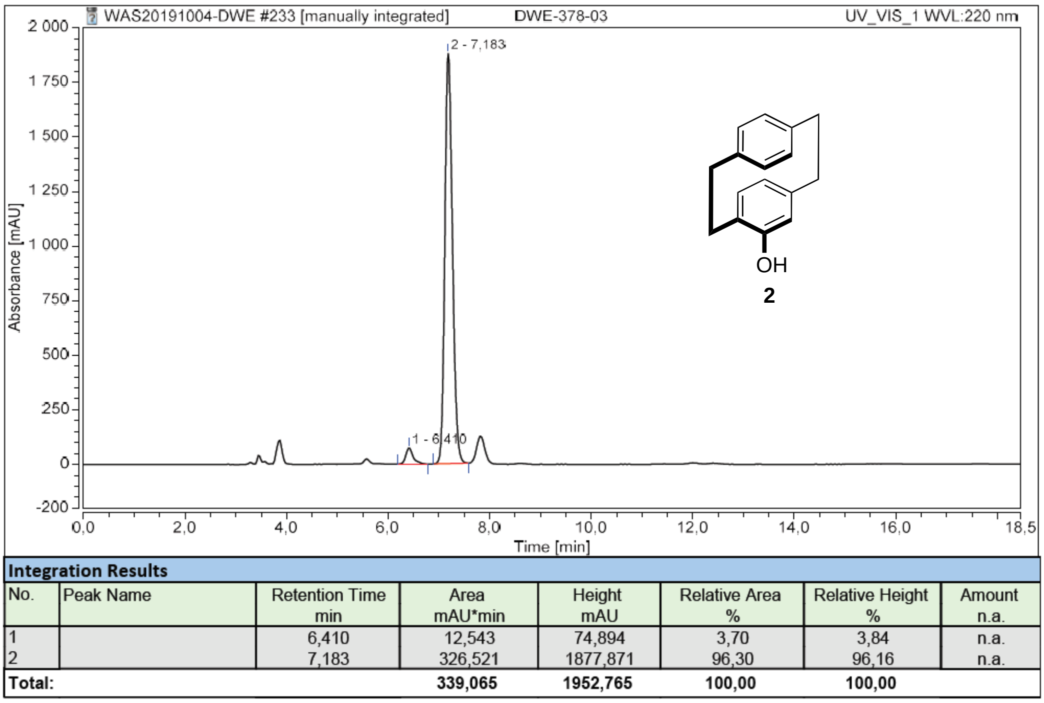

Integration Results

No.	Peak Name	Retention Time min	Area mAU*min	Height mAU	Relative Area %	Relative Height %	Amount n.a.
1		6,410	12,543	74,894	3,70	3,84	n.a.
2		7,183	326,521	1877,871	96,30	96,16	n.a.
**Total:**			**339,065**	**1952,765**	**100,00**	**100,00**	

HPLC chromatogram of (*rac*)-**3a**:

Chromatogram

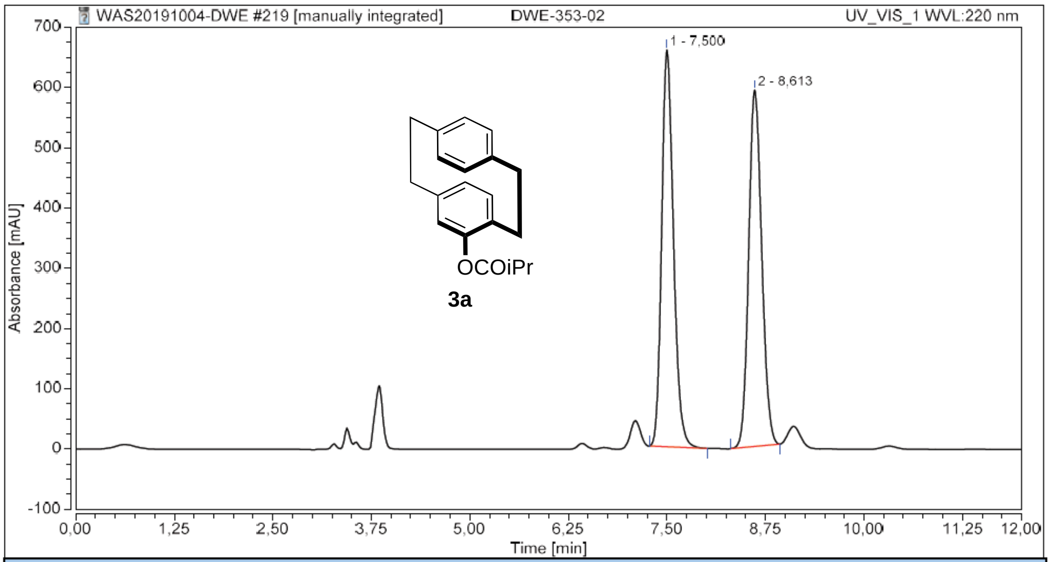

Integration Results

No.	Peak Name	Retention Time min	Area mAU*min	Height mAU	Relative Area %	Relative Height %	Amount n.a.
1		7,500	114,139	658,667	50,19	52,68	n.a.
2		8,613	113,277	591,646	49,81	47,32	n.a.
**Total:**			**227,415**	**1250,313**	**100,00**	**100,00**	

HPLC chromatogram of enantioenriched **3a**:

Chromatogram

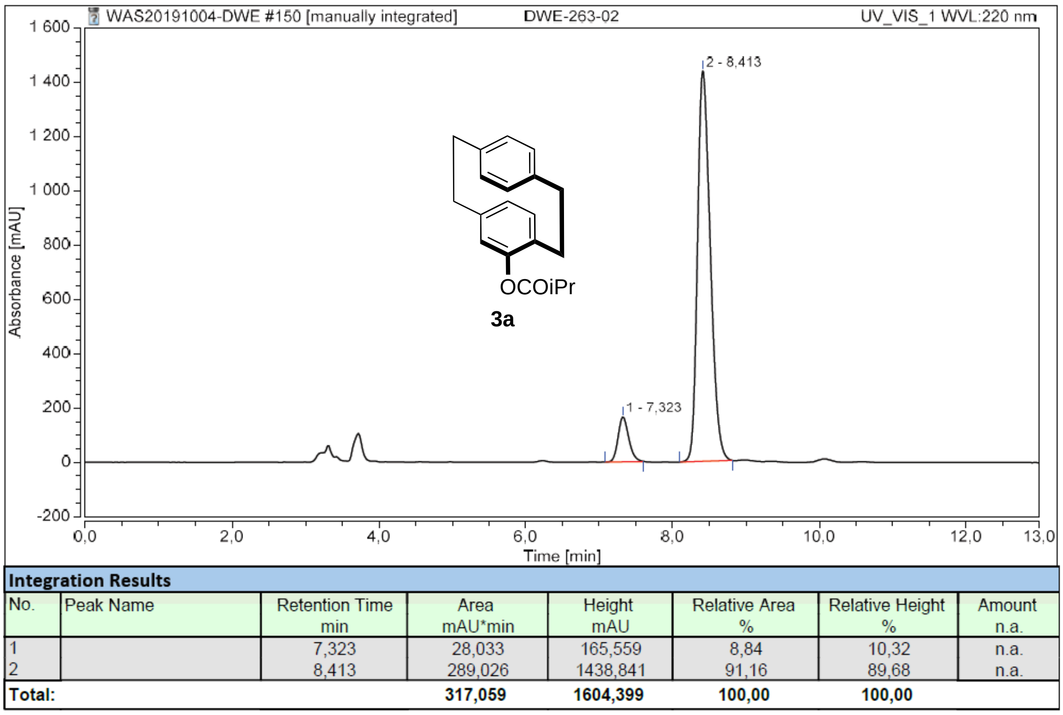

Integration Results

No.	Peak Name	Retention Time min	Area mAU*min	Height mAU	Relative Area %	Relative Height %	Amount n.a.
1		7,323	28,033	165,559	8,84	10,32	n.a.
2		8,413	289,026	1438,841	91,16	89,68	n.a.
**Total:**			**317,059**	**1604,399**	**100,00**	**100,00**	

HPLC chromatogram of (*rac*)-**3b**:

Chromatogram

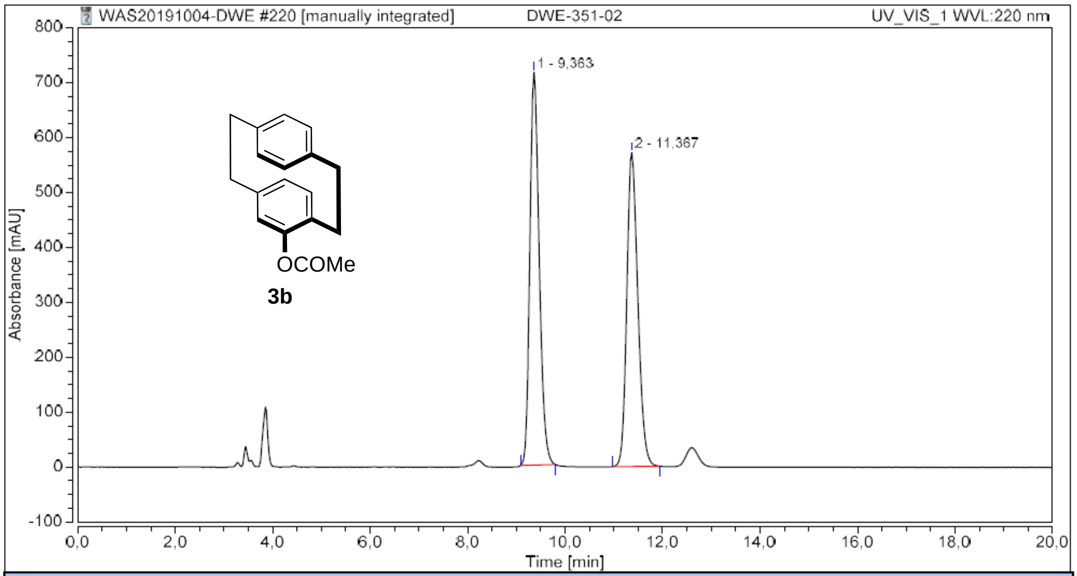

Integration Results

No.	Peak Name	Retention Time min	Area mAU*min	Height mAU	Relative Area %	Relative Height %	Amount n.a.
1		9,363	157,712	715,335	51,05	55,55	n.a.
2		11,367	151,216	572,495	48,95	44,45	n.a.
**Total:**			**308,928**	**1287,830**	**100,00**	**100,00**	

HPLC chromatogram of enantioenriched **3b**:

Chromatogram

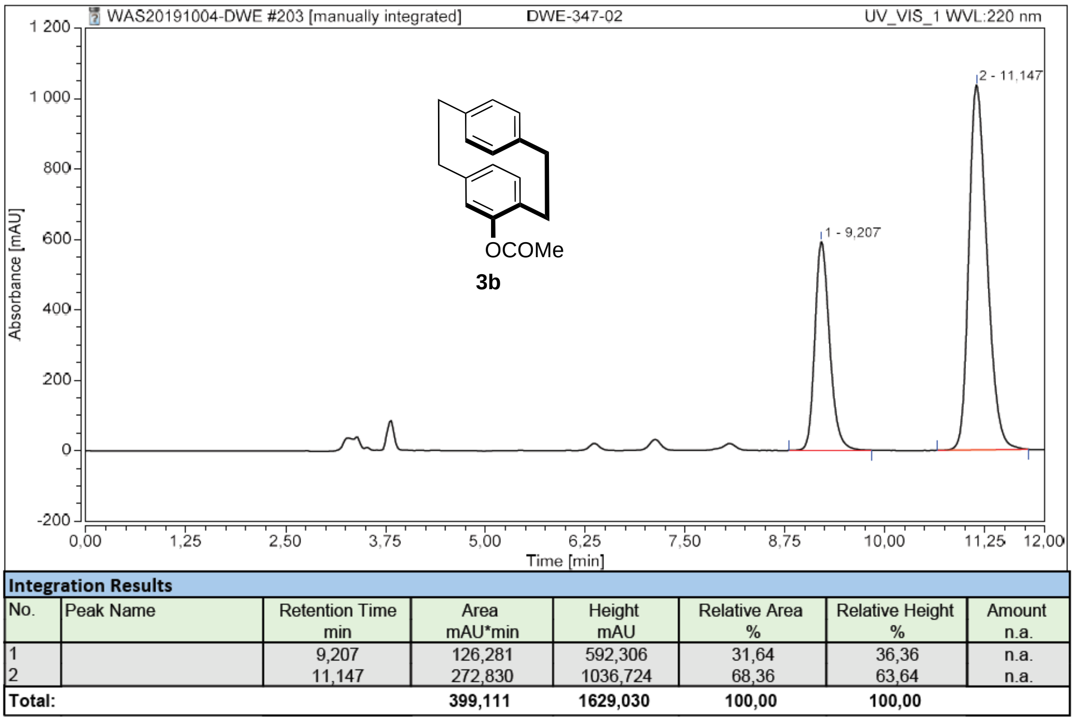

Integration Results

No.	Peak Name	Retention Time min	Area mAU*min	Height mAU	Relative Area %	Relative Height %	Amount n.a.
1		9,207	126,281	592,306	31,64	36,36	n.a.
2		11,147	272,830	1036,724	68,36	63,64	n.a.
**Total:**			**399,111**	**1629,030**	**100,00**	**100,00**	

HPLC chromatogram of (*rac*)-**3c**:

Chromatogram

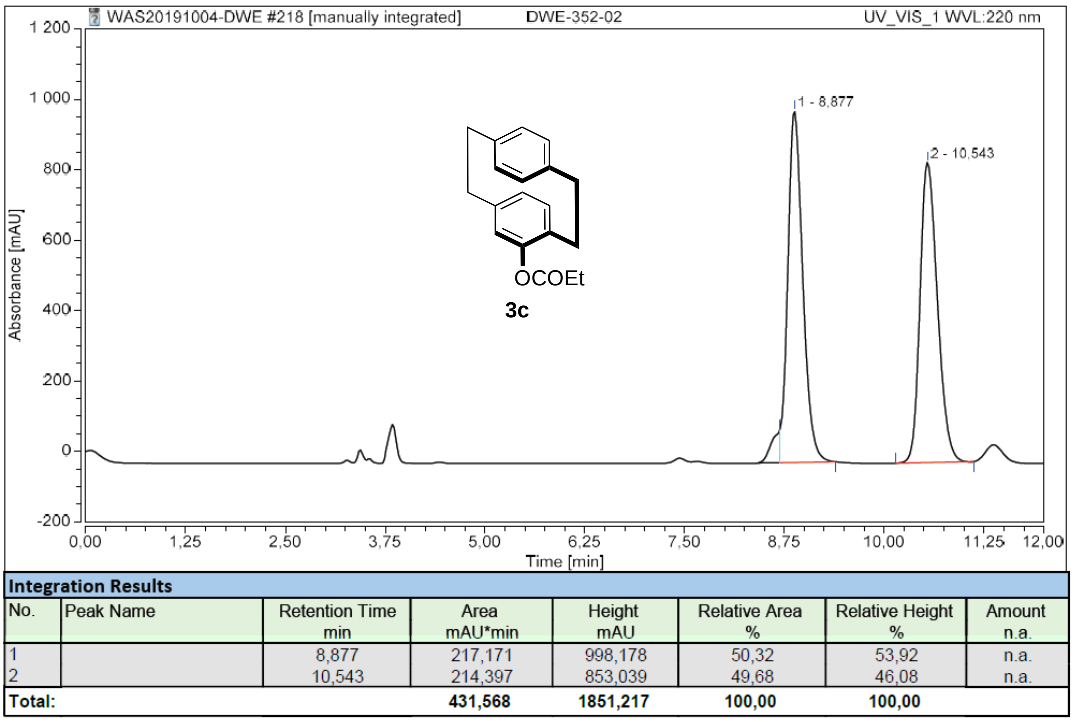

Integration Results

No.	Peak Name	Retention Time<br>min	Area<br>mAU*min	Height<br>mAU	Relative Area<br>%	Relative Height<br>%	Amount<br>n.a.
1		8,877	217,171	998,178	50,32	53,92	n.a.
2		10,543	214,397	853,039	49,68	46,08	n.a.
**Total:**			**431,568**	**1851,217**	**100,00**	**100,00**	

HPLC chromatogram of enantioenriched **3c**:

Chromatogram

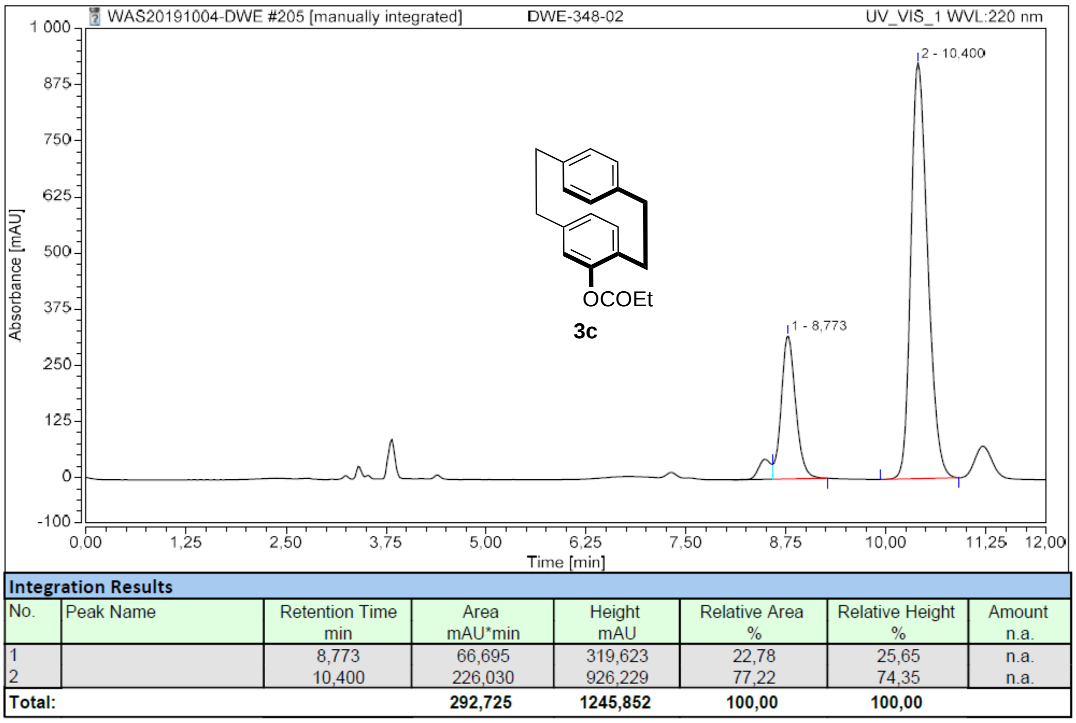

Integration Results

No.	Peak Name	Retention Time<br>min	Area<br>mAU*min	Height<br>mAU	Relative Area<br>%	Relative Height<br>%	Amount<br>n.a.
1		8,773	66,695	319,623	22,78	25,65	n.a.
2		10,400	226,030	926,229	77,22	74,35	n.a.
**Total:**			**292,725**	**1245,852**	**100,00**	**100,00**	

HPLC chromatogram of (*rac*)-**3d**:

Chromatogram

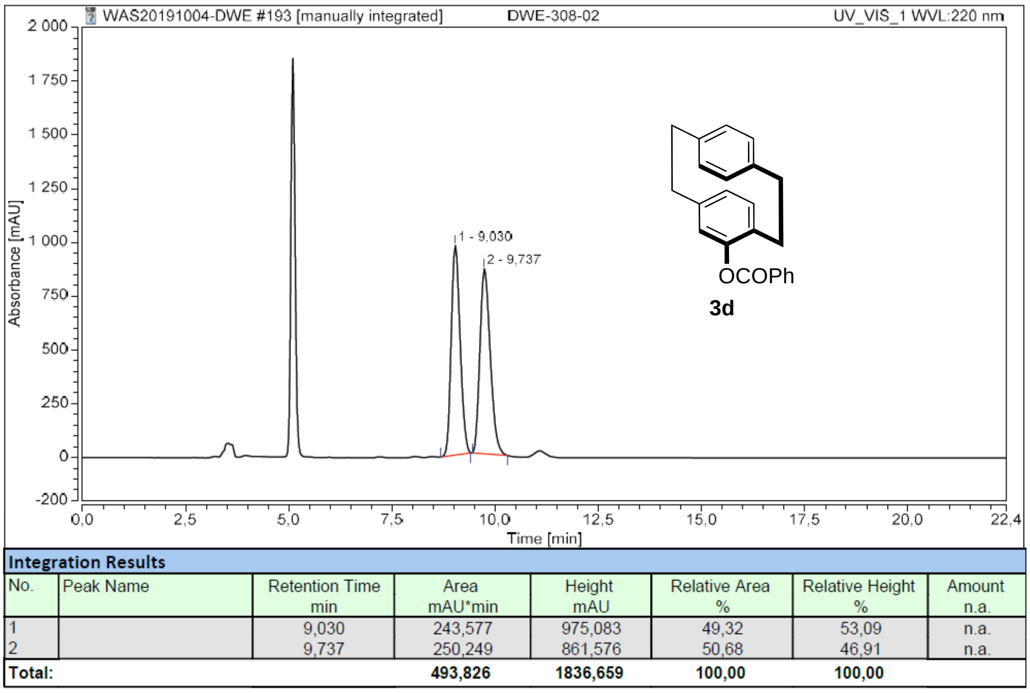

Integration Results

No.	Peak Name	Retention Time min	Area mAU*min	Height mAU	Relative Area %	Relative Height %	Amount n.a.
1		9,030	243,577	975,083	49,32	53,09	n.a.
2		9,737	250,249	861,576	50,68	46,91	n.a.
**Total:**			**493,826**	**1836,659**	**100,00**	**100,00**	
